# Supplementary material for: Sex-specific regulation of inhibition and network activity by local aromatase in the mouse hippocampus
Source: Nat Commun. 2022 Jul 7;13:3913. doi: 10.1038/s41467-022-31635-3 (PMC9262915; doi:10.1038/s41467-022-31635-3)
Supplement: Supplementary file 1 — Supplementary Information [file 41467_2022_31635_MOESM1_ESM.pdf]

Supplementary information

Sex-specific regulation of inhibition and network activity by local aromatase in the mouse hippocampus

Alicia Hernández-Vivanco[1#], Nuria Cano-Adamuz [1#], Alberto Sánchez-Aguilera [1,2], Alba González-Alonso[1], Alberto Rodríguez-Fernández[1], Íñigo Azcoitia [3], Liset Menéndez de la Prida[1], Pablo Méndez[1*]

Affiliation:
1) Instituto Cajal (CSIC)
Av Dr. Arce 37
28002
Madrid, Spain

2) Department of Physiology, Faculty of Medicine, Universidad Complutense de Madrid
IdISSC, Avda Complutense s/n
28040
Madrid, Spain

3) Department of Cell Biology, Universidad Complutense de Madrid
C José Antonio Nováis 12
28040
Madrid, Spain

[#] Equal contribution

* Corresponding author:

Pablo Méndez
Cajal Institute (CSIC)
Av Dr. Arce 37
28002
Madrid, Spain
pmendez@cajal.csic.es

Includes supplementary figures 1-7

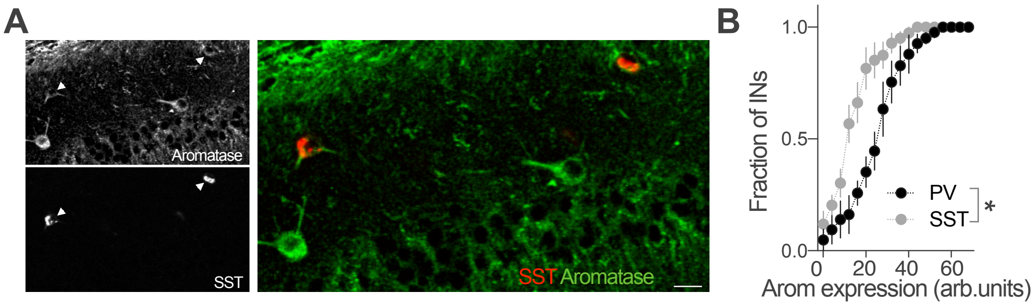

Fig. S1. Aromatase expression in female hippocampus CA1 INs, related to Fig. 1.

A. Simultaneous immunohistochemical detection of aromatase protein (green) and somatostatin (SST, red) in dorsal hippocampus CA1 region of female mice. Single-channel images are reproduced in grey scales in the left part of the panel. Arrowheads point to SST-INs in the *stratum oriens* of dorsal hippocampus CA1. Scale bar: 20 μm.

B. Graph shows frequency distribution analysis of Aromatase staining intensities in PV (black) and SST (grey) INs. Aromatase expression was higher in PV-INs compared with SST-INs. 2-way ANOVA, IN type F (1, 5) = 10.98, *, p = 0.021. n = 3 mice. Graph represents mean ± SEM. Source data are provided as a Source Data file.

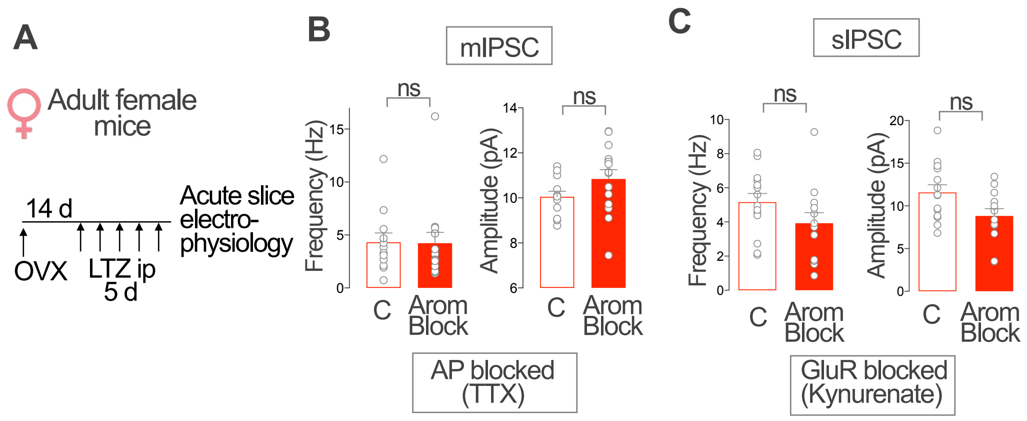

Fig. S2. *Aromatase blockade did not increase frequency of miniature mIPSCs (TTX insensitive) or sIPSCs recorded in the absence of glutamatergic transmission (Kynurenate insensitive), related to Fig. 3 and Fig. 4.*

A. Ovariectomized (OVX) adult female mice received daily intraperitoneal (ip) injections of the aromatase blocker letrozole (LTZ) or vehicle (C). On the fifth day of treatment, acute slices were prepared and electrophysiological recordings of sIPSCs performed from CA1 PYR neurons in the presence of the sodium channel blocker tetrodotoxin (TTX) to block action potentials (AP, B) or the glutamate receptor (GluR) blocker kynurenic acid (kynurenate, C).

B. Aromatase blockade failed to alter mIPSCs frequency. Recordings were performed under action potential blockade with TTX. Frequency, Mann Whitney test, $U = 76$, $p = 0.7$; amplitude, Mann Whitney test, $U = 49$, $p = 0.08$. $n = 12$, 14 cells from 3 mice per group.

C. Aromatase blockade-induced increase in sIPSCs frequency was not detected when recordings were performed under GluR blockade with kynurenate. Frequency, Mann Whitney test, U = 54, p = 0.07; amplitude, Mann Whitney test, $U = 53$, $p = 0.07$. $n = 14$, 13 cells from 3 mice per group.

Graphs represent mean ± SEM (columns and bars) and individual values (grey circles) for each experimental condition. ns $p > 0.05$. Source data are provided as a Source Data file.

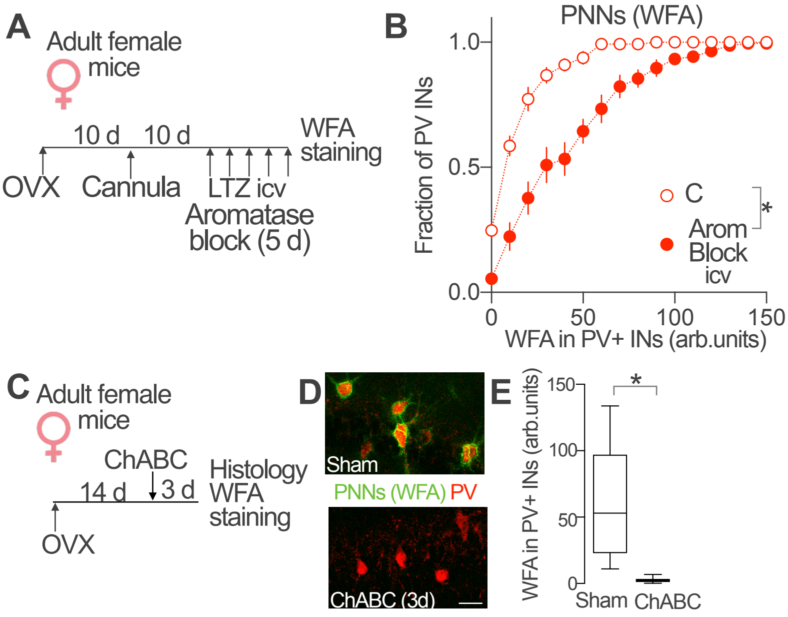

Fig. S3. ChABC degradation of PNNs enwrapping PV-INs in OVX female mice, related to Fig. 4.

A. Adult female mice were implanted with a guide cannula 10 days after ovariectomy (OVX). Ten days later, they received daily intracerebroventricular (icv) injections of vehicle (C) or the aromatase blocker LTZ for 5 days. At the end of the treatment, mice were sacrificed and processed for WFA staining.

B. Graph shows frequency distribution analysis of WFA staining intensities around PV+ INs. Intracerebroventricular aromatase blockade increases WFA staining intensity around OVX female CA1 PV-INs. 2-way ANOVA, Treatment F (1, 7) = 19.85, p = 0.003. n = 4, 5 mice. Graph represents mean ± SEM.

C. OVX female mice received a single intrahippocampal injection of Chondroitinase ABC (ChABC) or vehicle (Sham). WFA staining of CA1 PV-INs was assessed 3 days later.

D. Representative images of CA1 PV-IN in vehicle (Sham) and ChABC injected animals. ChABC treatment reduced WFA staining associated to PNNs. Scale bar 20µm.

E. Population data. ChABC treatment decreased WFA staining of CA1 PV-IN. Mann Whitney test, U = 104, p < 0.0001. n = 70, 74 neurons from 3 mice per group.

Whisker in plot represents median and 10-90 percentiles. * p < 0.05. Source data are provided as a Source Data file.

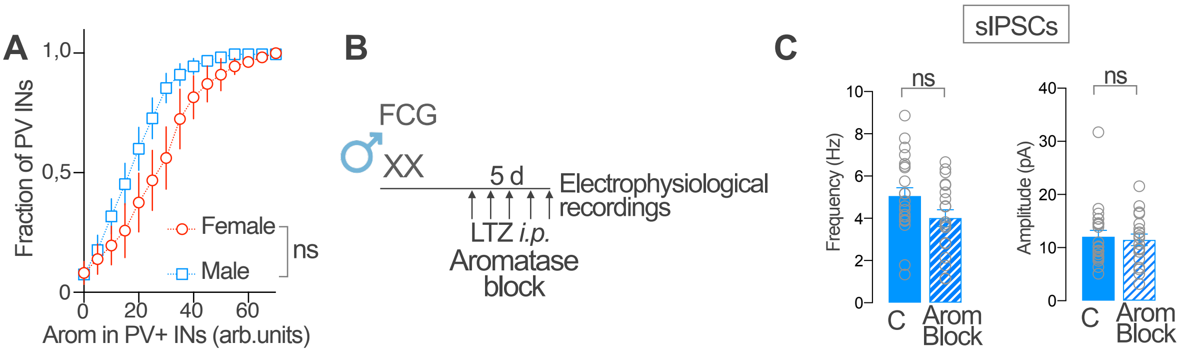

Fig. S4. Aromatase blockade failed to increase sIPSCs frequency in gonadal male mice with female sex chromosomes, related to Fig. 5.

A. Frequency distribution analysis of aromatase staining intensities in PV+ INs of male (blue) and intact female (red) mice. Aromatase staining intensity in CA1 PV IN did not differ in male and female mice. Two-way ANOVA, Sex F (1, 10) = 2.9, p = 0.12. n = 8, 4 mice. Graph represents mean ± SEM.

B. Male FCG with female XX sexual chromosomes mice received intraperitoneal (ip) injections of the aromatase blocker Letrozole for five days. On the fifth day of treatment, sIPSCs frequency and amplitude were determined in CA1 PYR neurons.

C. No difference was detected in the frequency or amplitude of sIPSCs in XX FCG male treated with aromatase blocker. Frequency, unpaired t-test, t (39) = 1.86, p = 0.07; Amplitude, unpaired t-test t (39) = 0.36, p = 0.72; n = 22, 19 cells from 3 animals per group. Graphs represent mean +/- SEM (columns and bars) and individual values (grey circles) for each experimental condition. ns p > 0.05. Source data are provided as a Source Data file.

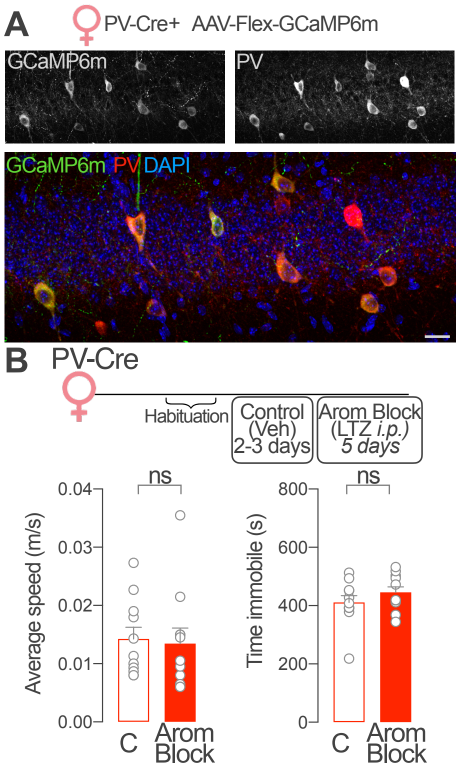

Fig. S5. Aromatase regulates female PV-IN activity in vivo, related to Fig. 6.

A. Representative immunostaining of CA1 PV-Cre female mice after infection with AAV-Flex-GCaMP6m. GCaMP6 (green) containing cells expressed the IN marker parvalbumin (PV, red)). Single-channel images are represented in grey scale in the upper part of the panel. Scale bar 20 microns.

B. Aromatase blockade failed to significantly alter female mice average exploration speed and immobility in an open field arena. No change exploration speed and immobility were detected in the Arom Block sessions with respect to control conditions. Average speed, paired two-tailed t-test, t (10) = 0.4634 p = 0.6530; time immobile paired, two-tailed t-test, t (10) = 1.399 p = 0,1920, n = 11 mice, ns p > 0.05. Graphs represent mean ± SEM. Source data are provided as a Source Data file.

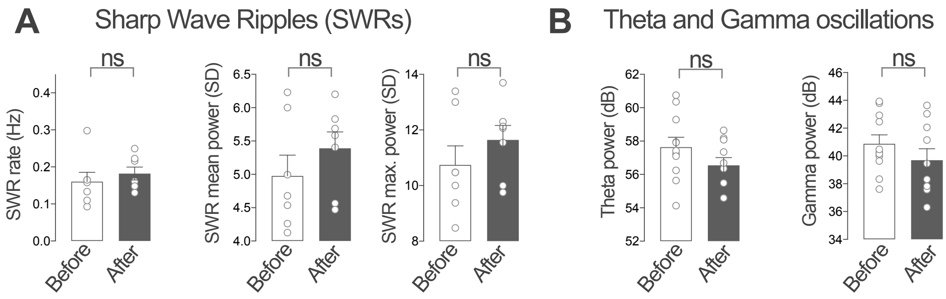

Fig. S6. No effect of repetitive recordings was observed on SWRs, gamma and theta oscillations in vehicle treated OVX mice, related to Fig. 7.

A. Hippocampal recordings were performed on OVX female mice before and after 5 days of vehicle treatment. No significant differences were observed in SWR rate, mean and maximum power despite repetitive penetrations. SWR rate, Mann Whitney test, $U = 19$, $p = 0.51$. SWR mean power, Mann Whitney test, $U = 18$, $p = 0.45$. SWR max. power, Mann Whitney test, $U = 18$, $p = 0.45$. $n = 7$ recordings from 5 mice.

B. As in A, no significant differences were observed in the power of gamma and theta oscillations. Theta power, Mann Whitney test, $U = 39$, $p = 0.27$. Gamma power, Mann Whitney test, $U = 36$, $p = 0.19$. $n = 10$, 11 recordings from 5 mice. Graphs represent mean +/- SEM (columns and bars) and individual values (grey circles) for each experimental condition. ns $p > 0.05$. Source data are provided as a Source Data file.

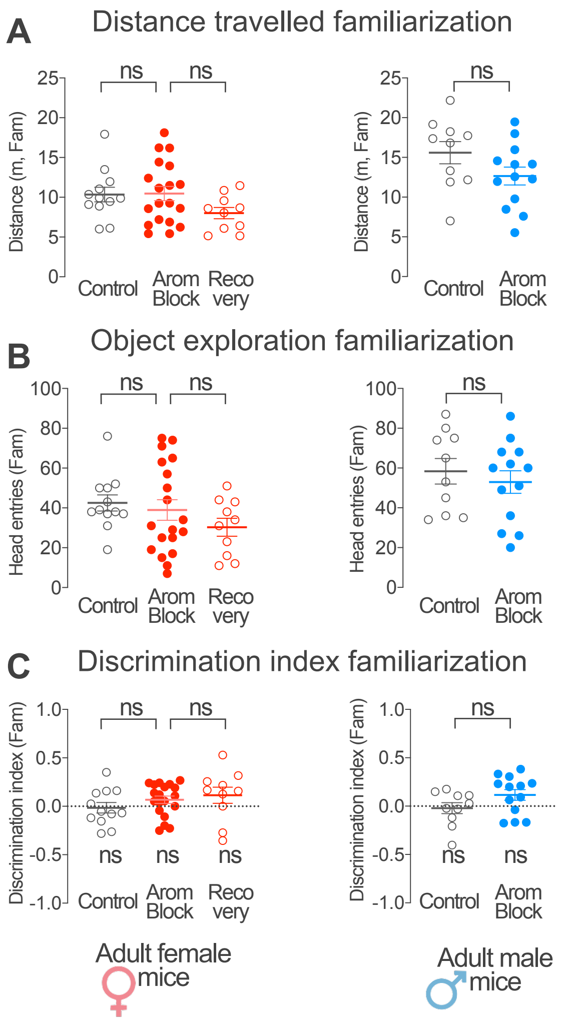

Fig. S7. Brain aromatase regulates hippocampal memory, related to Fig. 8.

A. Mice locomotion during the familiarization session of the NOL test. Values represent the total distance during the 10 min familiarization session. Female mice, one-way ANOVA: F (2, 38) = 1.94, p = 0.16; Bonferroni's comparison tests, C vs Arom Block p = 0.99, Arom Block vs Recovery p = 0.12. n = 12, 19, 10 mice. Male mice; unpaired two tailed t test, t (21) = 1.66, ns p = 0.11. n = 10, 13 mice per group.

B. Object exploration during the familiarization session of the NOL test. Values represent the total head entries in the object peripheral zone during the 10 min familiarization session. Female mice, one-way ANOVA: F (2, 38) = 1.23, p = 0.30; Bonferroni's comparison tests, C vs Arom Block p = 0.99, Arom Block vs Recovery p = 0.48. n = 12, 19, 10 mice. Male mice; unpaired two tailed t test, t (21) = 0.63, ns p = 0.53 n = 10, 13 mice per group.

C. Discrimination index during the familiarization (Fam) session. Discrimination index $\neq 0$ indicates preferential exploration of one object during the familiarization session. Dotted line represents chance levels. Female mice; one sample t tests (indicated below the graph): Control, t (11) = 0.29, p = 0.78, Arom Block, t (18) = 1.75, ns p = 0.10; Recovery, t (9) = 1.36, p = 0.21. One way ANOVA (indicated above the graph): F (2, 38) = 1.22, p = 0.31; Bonferroni's comparison tests, C vs Arom Block p = 0.47, Arom Block vs Recovery p = 0.55. n = 12, 19, 10 mice. Male mice; one sample t tests (indicated below the graph): Control, t (9) = 0.36, p = 0.72; Arom Block, t (12) = 2.90, p = 0.06. Unpaired two tailed t test (indicated above the graph), t (21) = 1.71, ns p = 0.10. n = 10, 13 mice per group.

Graphs represent mean +/- SEM (line and bars) and individual values (circles) for each experimental condition. ns $p > 0.05$. Source data are provided as a Source Data file.
