# Supplementary material for: Sex-specific regulation of inhibition and network activity by local aromatase in the mouse hippocampus
Source: Nat Commun. 2022 Jul 7;13:3913. doi: 10.1038/s41467-022-31635-3 (PMC9262915; doi:10.1038/s41467-022-31635-3)
Supplement: Supplementary file 3 — Reporting Summary [file 41467_2022_31635_MOESM3_ESM.pdf]

nature portfolio

Corresponding author(s): Pablo Méndez

Last updated by author(s): Jun 17, 2022

Reporting Summary

Nature Portfolio wishes to improve the reproducibility of the work that we publish. This form provides structure for consistency and transparency in reporting. For further information on Nature Portfolio policies, see our Editorial Policies and the Editorial Policy Checklist.

Statistics

For all statistical analyses, confirm that the following items are present in the figure legend, table legend, main text, or Methods section.

n/a	Confirmed	
☐	☒	The exact sample size (*n*) for each experimental group/condition, given as a discrete number and unit of measurement
☐	☒	A statement on whether measurements were taken from distinct samples or whether the same sample was measured repeatedly
☐	☒	The statistical test(s) used AND whether they are one- or two-sided<br>*Only common tests should be described solely by name; describe more complex techniques in the Methods section.*
☐	☒	A description of all covariates tested
☐	☒	A description of any assumptions or corrections, such as tests of normality and adjustment for multiple comparisons
☐	☒	A full description of the statistical parameters including central tendency (e.g. means) or other basic estimates (e.g. regression coefficient) AND variation (e.g. standard deviation) or associated estimates of uncertainty (e.g. confidence intervals)
☐	☒	For null hypothesis testing, the test statistic (e.g. *F*, *t*, *r*) with confidence intervals, effect sizes, degrees of freedom and *P* value noted<br>*Give P values as exact values whenever suitable.*
☒	☐	For Bayesian analysis, information on the choice of priors and Markov chain Monte Carlo settings
☒	☐	For hierarchical and complex designs, identification of the appropriate level for tests and full reporting of outcomes
☒	☐	Estimates of effect sizes (e.g. Cohen's *d*, Pearson's *r*), indicating how they were calculated

Our web collection on statistics for biologists contains articles on many of the points above.

Software and code

Policy information about availability of computer code

Data collection	Electrophysiological data was collected using Clampex v10.7.0.3 (Axon Instruments). Fiber photometry data was collected using Synapse software v90 (Tucker-Davies technologies). Behavioral data was collected using the AnyMaze software v7.10. Confocal microscopy images were acquired using the Leica software LAS AF v2.7.3.9723.
Data analysis	Analysis of electrophysiological and fiber photometry signals was implemented in MATLAB 2019a (MathWorks). IPSC were analyzed using pClamp 10.7.0.3 (Axon Instruments) and a custom written software (Detector, courtesy J. R. Huguenard, Stanford University), as previously described (Manseau et al., 2010). Detrending of fiber photometry signal was performed by an script script available from the manufacturer of the fiber photometry system (Tucker Davis). It can be found at Tucker Davis at https://www.tdt.com/docs/sdk/offline-data-analysis/offline-data-matlab/fiber-photometry-epoch-averaging-example/. The script used for illustration of fiber photometry signal alignment to locomotion/immobiliy transitions (Fig. 6D) is described in the Methods section. Image analysis was performed with Fiji (ImageJ) software v2.1.0/1.53c. Statistical analysis was performed using Prism 8 (Graphpad software).

For manuscripts utilizing custom algorithms or software that are central to the research but not yet described in published literature, software must be made available to editors and reviewers. We strongly encourage code deposition in a community repository (e.g. GitHub). See the Nature Portfolio guidelines for submitting code & software for further information.

Data

Policy information about availability of data

All manuscripts must include a data availability statement. This statement should provide the following information, where applicable:

- Accession codes, unique identifiers, or web links for publicly available datasets
- A description of any restrictions on data availability
- For clinical datasets or third party data, please ensure that the statement adheres to our policy

The raw data and the routines and codes that support the findings of the current study are available from the corresponding author upon reasonable request. Source data are provided with this paper.

Field-specific reporting

Please select the one below that is the best fit for your research. If you are not sure, read the appropriate sections before making your selection.

☒ Life sciences ☐ Behavioural & social sciences ☐ Ecological, evolutionary & environmental sciences

For a reference copy of the document with all sections, see nature.com/documents/nr-reporting-summary-flat.pdf

Life sciences study design

All studies must disclose on these points even when the disclosure is negative.

Sample size	No sample size calculation was performed. Sample size was determined before data collection based on information from previous studies.
Data exclusions	In fiber photometry photometry experiments, animals whose implant detached before the end of the complete protocol were removed from the analysis.
Replication	Drug effects were controlled in a group of animals treated with the vehicle of the tested drug, equal number of animals were used in experimental and control groups when possible. The effect of the pharmacological treatment with enzyme inhibitor was recovered with the enzyme product. Key experiments were replicated in two different mice strains (C57BL6 and CD1) of different genotypes. This include the efect of Letrozole in C57BL6 female mice, XX and XY CD1 female mice and the lack of effect of Letrozole in C57BL6 male mice and in CD1 XX males mice.<br>The experiments reported in Fig.1E,F were replicated three times, with similar results. The experiment in Fig. 1G was replicated twice, with similar results. The experiment in Fig. 2A-C was replicated twice with similar results. The experiment reported in Fig. 3A,B, D for intact C57Bl6 mice was replicated in intact CD1 mice (reported in Fig. 4F). The experiment presented in Fig. 4A, B was replicated twice with similar results. Moreover, intraperitoneal and intracerebral application of letrozole produced similar effects (Fig. S3A, B). Experiments reported in Fig. 5A, C for male C57Bl6 mice were replicated in male CD1 mice with similar results.
Randomization	Animals were randomly assigned to experimental groups.
Blinding	Investigators were not blind during data collection or analysis, equal parameter and protocols were consistently applied during the data acquisition and analysis. Blinding is not relevant to behavioral experiments since behavior was analyzed automatically without manual scoring.

Reporting for specific materials, systems and methods

We require information from authors about some types of materials, experimental systems and methods used in many studies. Here, indicate whether each material, system or method listed is relevant to your study. If you are not sure if a list item applies to your research, read the appropriate section before selecting a response.

Materials & experimental systems

n/a	Involved in the study	
☐	☒	Antibodies
☒	☐	Eukaryotic cell lines
☒	☐	Palaeontology and archaeology
☐	☒	Animals and other organisms
☒	☐	Human research participants
☒	☐	Clinical data
☒	☐	Dual use research of concern

Methods

n/a	Involved in the study	
☒	☐	ChIP-seq
☒	☐	Flow cytometry
☒	☐	MRI-based neuroimaging

Antibodies

Antibodies used	Parvalbumin (mouse monoclonal, code 235 (lot 10-11) and guinea pig polyclonal, code GP42 (both from Swant), aromatase (1:1000, in-house production), SATB1 (mouse monoclonal, C-6, code sc-376096, Lot E0517, Santa Cruz Biotechnologies) and SST (rat monoclonal, code MAB354, Lot 3205921 Millipore). Alexa conjugated secondary antibodies from Abcam: Goat Anti-Mouse IgG Alexa 488, code 150113; Goat Anti-Rabbit IgG Alexa 488, code 150077, Goat Anti-Guinea pig IgG Alexa 647, code 150187; Goat Anti-Rabbit IgG Alexa 568, code 175471. For biotinylated WFA, we used Streptavidin Alexa 488 conjugated also from Abcam (code S11223).
Validation	Validation of commercial antibodies is described by the respective manufacturers . Parvalbumin antibody Code 235 has been validated in free-floating sections using the parvalbumin knock-out mice. Parvalbumin antibody code GP72 has been validated using recombinant parvalbumin protein using inmunoblots and does not stain the brain of parvalbumin knock-out mice. SATB1 monoclonal antibody recognizes SATB1 protein in inmunoblots and on fixed tissue and has been succesfully used to stain brain sections (PMID: 30811984). We and others have successfully used MAB354 to detect STT protein in brain slices (PMID: 31548723, PMID: 34845987, PMID: 26875623). The validation of Aromatase antibody is described in the reference Yague et al 2006 (PMID: 16426763).

Animals and other organisms

Policy information about studies involving animals; ARRIVE guidelines recommended for reporting animal research

Laboratory animals	C57BL/6J wild type, PV-Cre (Pvalb tm1(cre)Arbr/J) and Four Core Genotype mice (FCG, B6.Cg-Tg(Sry)2Ei Srydl1Rlb/ArnoJ) were used in the study. PV-Cre and FCG mice were maintained in a C57BL/6J and CD1 genetic background, respectively. Experiments were performed in males and females from both PV-Cre and FCG mice. Sex of the animals is described for each experiment. All animals were 8-14 weeks during the experiments.
Wild animals	The study did not involve wild animals.
Field-collected samples	The study did not involve field-collected samples.
Ethics oversight	Experiments were performed according to protocols approved by the Institutional Animal Care and Use Committee of the Cajal Institute and approved by local veterinary office (Comunidad de Madrid, Spain).

Note that full information on the approval of the study protocol must also be provided in the manuscript.

March 2021
